# Incidence of Second Malignancies for Prostate Cancer

**DOI:** 10.1371/journal.pone.0102596

**Published:** 2014-07-21

**Authors:** Mieke Van Hemelrijck, Anita Feller, Hans Garmo, Fabio Valeri, Dimitri Korol, Silvia Dehler, Sabine Rohrmann

**Affiliations:** 1 Cancer Epidemiology Unit, Division of Cancer Studies, School of Medicine, King's College London, London, United Kingdom; 2 Foundation National Institute for Cancer Epidemiology and Registration (NICER), Zurich, Switzerland; 3 Institute of Social and Preventive Medicine, University of Bern, Bern, Switzerland; 4 Cancer Registry Zurich and Zug, Zurich, Switzerland; 5 Institute of Social and Preventive Medicine, University of Zurich, Zurich, Switzerland; National Health Research Institutes, Taiwan

## Abstract

**Introduction:**

There is a need to assess risk of second primary cancers in prostate cancer (PCa) patients, especially since PCa treatment may be associated with increased risk of second primary tumours.

**Methods:**

We calculated standardized incidence ratios (SIRs) for second primary tumours comparing men diagnosed with PCa between 1980 and 2010 in the Canton of Zurich, Switzerland (n = 20,559), and the general male population in the Canton.

**Results:**

A total of 1,718 men developed a second primary tumour after PCa diagnosis, with lung and colon cancer being the most common (15 and 13% respectively). The SIR for overall second primary cancer was 1.11 (95%CI: 1.06–1.17). Site-specific SIRs varied from 1.19 (1.05–1.34) to 2.89 (2.62–4.77) for lung and thyroid cancer, respectively. When stratified by treatment, the highest SIR was observed for thyroid cancer (3.57 (1.30–7.76)) when undergoing surgery, whereas liver cancer was common when treated with radiotherapy (3.21 (1.54–5.90)) and kidney bladder was most prevalent for those on hormonal treatment (3.15 (1.93–4.87)). Stratification by time since PCa diagnosis showed a lower risk of cancer for men with PCa compared to the general population for the first four years, but then a steep increase in risk was observed.

**Conclusion:**

In the Canton of Zurich, there was an increased risk of second primary cancers among men with PCa compared to the general population. Increased diagnostic activity after PCa diagnosis may partly explain increased risks within the first years of diagnosis, but time-stratified analyses indicated that increased risks remained and even increased over time.

## Introduction

The number of men living with prostate cancer (PCa) is growing rapidly. In the US, currently more than two million men are PCa survivors as the disease can often be treated successfully [1]. In Switzerland the prevalence rate of PCa was estimated to be 580.5 per 100,000 in 2010, accounting for 45,421 men living with the disease [2]. It is therefore of public health importance to assess the risk of second primary cancers in these men, especially since it is thought that PCa treatment may also be associated with an increased risk of second primary tumours [3]–[7].

A Swedish study based on the National Prostate Cancer Register (NPCR) concluded that about 17% of all PCa occurred in combination with another primary cancer (before or after PCa diagnosis) [8]. A study based on the Israel Cancer Registry, including 29,593 men diagnosed with PCa, found an increased risk of rectal cancer among men treated with radiation therapy compared to those managed by surgery [9]. However, a recent retrospective cohort study of 24,038 US men diagnosed with localized PCa did not find an association between androgen deprivation therapy (ADT) and risk of second primary cancers [3].

Apart from the potential risk of adverse treatment effects, it is also of interest to assess the risk of second primary cancers in men with PCa to identify potential underlying etiological mechanisms [8], [10]. Given the increasing evidence for an association between Insulin-like Growth Factor (IGF)-I and risk of PCa, the Swedish PCBaSE study evaluated the risk of other IGF-I related cancers (colon, rectum, thyroid, and hematologic cancers) and found an increased risk among those men treated with ADT [8].

To further disentangle the association between PCa and risk of second primary cancers we therefore calculated the standardized incidence ratios (SIRs) comparing men diagnosed with PCa between 1980 and 2010 and the general male population in the canton of Zurich, Switzerland, while also taking into account treatment information.

## Methods

### Study population

The index cohort for this study included all men diagnosed with primary PCa between 1980 and 2010 in the canton of Zurich, Switzerland, as registered by the Cancer Registry Zurich and Zug (n = 20,559) [11]. None of the cancers were diagnosed at autopsy or co-diagnosed with another type of cancer. The Cancer Registry Zurich and Zug covers more than 1.3 million inhabitants so that it encompasses about one third of all registered cancer cases in Switzerland. All cases are registered based on the International Classification of Disease for Oncology (ICD-O) together with the basis of diagnosis (i.e. histology, autopsy, clinical investigation). The Cancer Registry confirmed complete and systematic matching of incident cases and death records as of 1997, but for the period prior to 1997 this distinction could not be determined for the canton of Zurich. Coverage of the Registry is almost complete, though there is some under-registration due to insufficient legal regulation of cancer registration on cantonal level for the years 2007–2010 [11]. The percentage death certificate only cases (DCO%) is estimated at 2.8% for the period 1997–2010 and the percentage of morphologically verified cases (MV%) is 93.9% for the period 1980–2010 [12]. The Cancer Registry also includes data on date of diagnosis, age at diagnosis, tumour grade, mode of detection, primary treatment, civil status as well as date of death. Diagnoses of first primary cancer after PCa were also taken from the Cancer Registry [11]. These were categorized according to the Eurostat European shortlist for cause of death [13].

In addition to overall cancer, we also studied those specific cancer types. The comparison cohort or background population consisted of all men alive in Zurich between 1980 and 2010, divided into 5-year age-categories ending at 85+.

### Statistical Methods

Since the Cancer Registry of Zurich is based on the entire Zurich population it was possible to calculate standardized incidence ratios (SIRs) to compare the risk of a second primary tumour in the PCa patient group with the risk of a primary tumour in the male population of the canton of Zurich. The SIR is defined as the ratio of the observed numbers of primary tumours to the expected numbers. The observed number of primary tumours was counted among the index cohort by age (5-year categories) and calendar-time categories (split per year). The expected number of primary tumours was counted among the comparison cohort by multiplying the time of follow-up with the corresponding age- and period-specific incidence rates. The time of follow-up was taken from the observed numbers and the age- and period-specific incidence rates (5-year age categories and per year calendar time) were defined as the number of cases in the comparison cohort divided by the corresponding person-time in this cohort. The 95% confidence intervals for the SIRs were estimated by assuming that the observed cases had a Poisson distribution using Byar's normal approximation [14], [15]. We performed stratified analyses by primary treatment, grade, year of diagnosis, and time since PCa diagnosis. For the latter analysis we performed an additional splitting of the data by follow-up time (0.5–1 year, 2 years, 3 years, 5years, and more than 10 years).To address detection bias we also repeated the above analyses for all urological cancers combined (kidney, bladder, testis, and penile cancers). To evaluate potential etiological mechanisms, we performed all analyses for all IGF-1 associated cancers (colon, rectum, thyroid, and haematological cancers) [16]. Finally, we conducted a sensitivity analysis whereby we excluded those tumours that occurred within 6 months of diagnosis of the first primary tumour. The latter addresses potential diagnostic bias and takes into account the difference between synchronous and metachronous cancers [17]. Statistical analyses were performed with Statistical Analysis Systems (SAS) release 9.3 (SAS Institute, Cary, NC).

## Results

The majority of men diagnosed with PCa were over 65 years old (76%) and had a grade II tumour (45%). A more detailed overview of patient characteristics is shown in Table 1. A total of 1,718 men developed a second primary tumour after PCa diagnosis, with lung and colon cancer being the most common (15 and 13%, respectively, Table 2).

**Table 1:** Baseline characteristics of men with primary PCa in the canton of Zurich, Switzerland, 1980–2010.

	All men with PCa (N = 20,559)	PCa men with second primary tumour (N = 1,718)	PCa men without second primary tumour (N = 18,841)
**Age** (years)			
Mean (SD)	71.65 (9.20)	71.35 (7.68)	71.67 (9.33)
<65	5018 (24.41)	328 (19.09)	4690 (24.89)
65–74	8076 (39.28)	848 (49.36)	7228 (38.36)
75–84	5904 (28.72)	480 (27.94)	5424 (28.79)
85+	1561 (7.59)	62 (3.61)	1499 (7.96)
**Civil status**			
Married/Partnership	11511 (55.99)	1112 (64.73)	10399 (55.19)
Single/Widowed/Divorced/Separated	4146 (20.17)	372 (21.65)	3774 (20.03)
Others/Unknown	4902 (23.84)	234 (13.62)	4668 (24.78)
**PCa Grade**			
I	3724 (18.11)	464 (27.01)	3260 (17.30)
II	9233 (44.91)	788 (45.87)	8445 (44.82)
III	5279 (25.68)	307 (17.87)	4987 (26.47)
IV	15 (0.00)	159 (9.25)	2149 (11.41)
Unknown/Missing	2308 (11.11)		
**Primary PCa Treatment**			
Surgery	5381 (26.17)	554 (32.25)	4827 (25.62)
Radiotherapy	1577 (7.67)	179 (10.42)	1398 (7.42)
Androgen Deprivation therapy	3194 (15.54)	288 (16.76)	2906 (15.42)
Missing or Surveillance	10407 (50.62)	697 (40.57)	9710 (51.54)
**Year of diagnosis**			
<1995	7520 (36.58)	797 (46.39)	6723 (35.68)
1995+	13039 (63.42)	921 (53.61)	12118 (64.32)

**Table 2:** The number of primary cancers diagnosed after primary PCa diagnosis.

ICD-10 Code	Site	N of primary cancers (%) (N = 1,718)
C00-14	Lip, oral cavity, pharynx	42 (2.4)
C15	Oesophagus	32 (1.9)
C16	Stomach	70 (4.1)
C18	Colon	216 (12.6)
C20-21	Rectum, anus	78 (4.5)
C22	Liver, intrahepatic bile ducts	47 (2.7)
C23-24	Gallbladder, biliary tract	21 (1.2)
C25	Pancreas	79 (4.6)
C32-34	Larynx, trachea/bronchus/lung	262 (15.3)
C62	Testis	2 (0.1)
C64	Kidney, renal cell	86 (5.0)
C67	Bladder	197 (11.5)
C43	Melanoma of the skin	108 (6.3)
C44	Non-melanoma of the skin	9 (0.5)
C71-72	Brain, nervous tissue	27 (1.6)
C73	Thyroid gland	15 (0.9)
C82-85	Non-Hodgkin lymphoma	107 (6.2)
C91-95	Leukemia	76 (4.4)
C81	Hodgkin lymphoma	3 (0.2)
C90	Multiple myeloma	58 (3.4)
*	Other cancers	183 (10.7)

Table 3 shows the SIRs for specific cancer types. Overall, there was an increased risk of a second primary tumour among men with PCa, compared to the general population (SIR: 1.11 (95%CI: 1.06–1.17)). Site-specific SIRs varied from 1.07 (95%CI: 1.05–1.34) to 2.89 (95%CI: 2.62–4.77) for testicular and thyroid cancer, respectively (Table 3).

**Table 3:** Standardized Incidence Ratios (SIRs) and 95% confidence intervals (CI) for primary cancer diagnosed after primary PCa diagnosis.

Type	Observed	Expected	SIR	95%CI	
All	1718	1545.67	1.11	1.06	1.17
lip	42	31.45	1.34	0.96	1.81
oesophagus	32	20.81	1.54	1.05	2.17
stomach	70	53.59	1.31	1.02	1.65
colon	216	124.66	1.73	1.51	1.98
anus	78	55.54	1.40	1.11	1.75
liver	47	31.65	1.49	1.09	1.97
galbladder	21	13.57	1.55	0.96	2.37
pancreas	79	46.12	1.71	1.36	2.13
lung	262	219.94	1.19	1.05	1.34
testis	2	1.87	1.07	0.12	3.85
kidney	86	35.70	2.41	1.93	2.98
bladder	197	94.26	2.09	1.81	2.40
melanoma	108	60.39	1.79	1.47	2.16
brain	27	16.22	1.66	1.10	2.42
thyroid	15	5.19	2.89	1.62	4.77
non-Hodgkin lymphomas	107	53.28	2.01	1.65	2.43
leukemia	76	41.71	1.82	1.44	2.28
Urology*	304	144.55	2.10	1.87	2.35
IGF**	416	305.55	1.36	1.23	1.50

* includes bladder, kidney, testis, and penile cancers.

** includes colon, rectum, thyroid, and haematological cancers.

When stratified by treatment, the highest SIR was observed for thyroid cancer (3.57 (95%CI: 1.30–7.76)) when undergoing surgery, whereas liver cancer was most common when treated with radiotherapy (3.21 (95%CI: 1.54–5.90)) and kidney bladder was most prevalent for those on hormonal treatment (3.15 (95%CI: 1.93–4.87)) (Table 4).

**Table 4:** Standardized Incidence Ratios (SIRs) and 95% confidence intervals (CI) for primary cancer diagnosed after primary PCa diagnosis, stratified by primary treatment.

	Surgery					Radiotherapy					ADT					Surveillance/Missing				
Type	Observed	Expected	SIR	95%CI		Observed	Expected	SIR	95%CI		Observed	Expected	SIR	95%CI		Observed	Expected	SIR	95%CI	
**All**	554	483.16	**1.15**	**1.05**	**1.25**	179	150.34	**1.19**	**1.02**	**1.38**	288	295.57	0.97	0.87	1.09	697	616.61	**1.13**	**1.05**	**1.22**
**lip**	16	8.98	**1.78**	**1.02**	**2.89**	5	3.20	1.56	0.50	3.65	4	5.29	0.76	0.20	1.94	17	13.98	1.22	0.71	1.95
**oesophagus**	6	5.93	1.01	0.37	2.20	3	2.15	1.39	0.28	4.07	5	3.18	1.57	0.51	3.66	18	9.53	**1.89**	**1.12**	**2.98**
**stomach**	24	18.40	1.30	0.84	1.94	8	4.55	1.76	0.76	3.47	18	12.37	1.45	0.86	2.30	20	18.28	1.09	0.67	1.69
**colon**	69	39.92	**1.73**	**1.34**	**2.19**	17	12.14	1.40	0.82	2.24	31	23.90	1.30	0.88	1.84	99	48.69	**2.03**	**1.65**	**2.48**
**anus**	28	17.41	**1.61**	**1.07**	**2.32**	7	5.37	1.30	0.52	2.69	13	10.64	1.22	0.65	2.09	30	22.12	1.36	0.91	1.94
**liver**	15	9.67	1.55	0.87	2.56	10	3.12	**3.21**	**1.54**	**5.90**	5	5.83	0.86	0.28	2.00	17	13.03	1.30	0.76	2.09
**galbladder**	13	4.30	**3.03**	**1.61**	**5.18**	3	1.25	2.40	0.48	7.00	1	2.65	0.38	0.00	2.10	4	5.37	0.74	0.20	1.91
**pancreas**	30	14.81	**2.03**	**1.37**	**2.89**	7	4.38	1.60	0.64	3.30	13	8.85	1.47	0.78	2.51	29	18.08	**1.60**	**1.07**	**2.30**
**lung**	86	69.71	**1.23**	**0.99**	**1.52**	17	21.98	0.77	0.45	1.24	48	42.51	1.13	0.83	1.50	111	85.74	**1.29**	**1.07**	**1.56**
**testis**	1	0.53	1.90	0.02	10.56	0	0.22				0	0.25				1	0.88	1.14	0.01	6.34
**kidney**	24	10.97	**2.19**	**1.40**	**3.25**	10	3.65	**2.74**	**1.31**	**5.04**	20	6.34	**3.15**	**1.93**	**4.87**	32	14.74	**2.17**	**1.49**	**3.07**
**bladder**	64	32.41	**1.97**	**1.52**	**2.52**	23	8.72	**2.64**	**1.67**	**3.96**	53	20.83	**2.54**	**1.91**	**3.33**	57	32.30	**1.76**	**1.34**	**2.29**
**melanoma**	25	16.97	1.47	0.95	2.17	13	6.49	**2.00**	**1.07**	**3.42**	11	8.75	1.26	0.63	2.25	59	28.18	**2.09**	**1.59**	**2.70**
**brain**	13	4.72	**2.76**	**1.47**	**4.71**	1	1.77	0.57	0.01	3.15	2	2.66	0.75	0.08	2.71	11	7.07	1.55	0.78	2.78
**thyroid**	6	1.68	**3.57**	**1.30**	**7.76**	1	0.48	2.08	0.03	11.55	2	1.10	1.82	0.20	6.57	6	1.93	**3.11**	**1.14**	**6.78**
**non-Hodgkin**	31	16.65	**1.86**	**1.26**	**2.64**	12	5.40	**2.22**	**1.15**	**3.88**	13	9.82	1.32	0.70	2.26	51	21.42	**2.38**	**1.77**	**3.13**
**leukemia**	24	13.54	**1.77**	**1.14**	**2.64**	10	3.91	**2.56**	**1.23**	**4.71**	10	8.12	1.23	0.59	2.27	32	16.15	**1.98**	**1.36**	**2.80**
**urology**	96	48.16	**1.99**	**1.61**	**2.43**	34	13.73	**2.48**	**1.71**	**3.46**	79	30.03	**2.63**	**2.08**	**3.28**	95	52.62	**1.81**	**1.46**	**2.21**
**IGF**	134	96.78	**1.38**	**1.16**	**1.64**	37	29.86	1.24	0.87	1.71	59	58.29	1.01	0.77	1.31	186	120.61	**1.54**	**1.33**	**1.78**

Stratification by tumour grade showed the highest SIR for non-Hodgkin lymphoma (3.05 (95%CI: 2.13–4.14)) for men with a grade I tumour, whereas for men with a grade II tumour the SIR was highest for thyroid cancer (3.34 (95%CI: 1.44–6.58, Table 5). Kidney cancer showed the highest statistically significant SIR for men with a grade III/IV tumour (2.56 (95%CI: 1.52–4.05)).

**Table 5:** Standardized Incidence Ratios (SIRs) and 95% confidence intervals (CI) for primary cancer diagnosed after primary PCa diagnosis, stratified by tumour grade.

	Grade I					Grade II					Grade III/IV					Grade Missing/Unknown				
Type	Observed	Expected	SIR	95%CI		Observed	Expected	SIR	95%CI		Observed	Expected	SIR	95%CI		Observed	Expected	SIR	95%CI	
**All**	464	362.4776	1.28	1.17	1.40	788	718.95	1.10	1.02	1.18	307	299.29	1.03	0.91	1.15	159	164.96	0.96	0.82	1.13
**lip**	12	7.060198	1.70	0.88	2.97	18	15.02	1.20	0.71	1.89	9	6.34	1.42	0.65	2.70	3	3.03	0.99	0.20	2.89
**oesophagus**	7	4.471422	1.57	0.63	3.23	17	9.88	1.72	1.00	2.76	6	4.42	1.36	0.50	2.96	2	2.04	0.98	0.11	3.54
**stomach**	17	13.68346	1.24	0.72	1.99	29	23.93	1.21	0.81	1.74	17	9.57	1.78	1.03	2.84	7	6.41	1.09	0.44	2.25
**colon**	52	29.86845	1.74	1.30	2.28	106	57.32	1.85	1.51	2.24	34	23.74	1.43	0.99	2.00	24	13.72	1.75	1.12	2.60
**anus**	23	13.17821	1.75	1.11	2.62	38	25.76	1.48	1.04	2.03	13	10.70	1.22	0.65	2.08	4	5.91	0.68	0.18	1.73
**liver**	9	7.385637	1.22	0.56	2.31	24	14.74	1.63	1.04	2.42	11	6.23	1.77	0.88	3.16	3	3.29	0.91	0.18	2.67
**galbladder**	8	3.223771	2.48	1.07	4.89	9	6.21	1.45	0.66	2.75	2	2.60	0.77	0.09	2.77	2	1.53	1.30	0.15	4.71
**pancreas**	29	10.93454	2.65	1.78	3.81	27	21.11	1.28	0.84	1.86	15	8.92	1.68	0.94	2.77	8	5.15	1.55	0.67	3.06
**lung**	69	52.72795	1.31	1.02	1.66	124	102.05	1.22	1.01	1.45	47	42.04	1.12	0.82	1.49	22	23.12	0.95	0.60	1.44
**testis**	0	0.414327	--	--	--	0	--	--	--	--	2	0.39	5.18	0.58	18.72	0	--	--	--	--
**kidney**	23	8.305704	2.77	1.75	4.16	38	16.73	2.27	1.61	3.12	18	7.02	2.56	1.52	4.05	7	3.64	1.92	0.77	3.96
**bladder**	56	24.02255	2.33	1.76	3.03	88	42.25	2.08	1.67	2.57	38	16.89	2.25	1.59	3.09	15	11.10	1.35	0.76	2.23
**melanoma**	22	12.90379	1.70	1.07	2.58	60	28.83	2.08	1.59	2.68	17	12.93	1.31	0.77	2.11	9	5.73	1.57	0.72	2.98
**brain**	10	3.680685	2.72	1.30	5.00	11	7.80	1.41	0.70	2.52	3	3.23	0.93	0.19	2.72	3	1.51	1.99	0.40	5.80
**thyroid**	3	1.277168	2.35	0.47	6.86	8	2.40	3.34	1.44	6.58	3	0.95	3.15	0.63	9.19	1	0.56	1.78	0.02	9.90
**non-Hodgkin**'**s lymphoma**	38	12.59427	3.02	2.13	4.14	49	24.73	1.98	1.47	2.62	15	10.32	1.45	0.81	2.40	5	5.64	0.89	0.29	2.07
**leukemia**	18	10.01195	1.80	1.06	2.84	33	18.99	1.74	1.20	2.44	11	7.93	1.39	0.69	2.48	14	4.77	2.93	1.60	4.92
**urology**	86	35.79827	2.40	1.92	2.97	132	65.71	2.01	1.68	2.38	61	26.65	2.29	1.75	2.94	25	16.40	1.52	0.99	2.25
**IGF**	116	72.42683	1.60	1.32	1.92	201	140.97	1.43	1.24	1.64	65	58.88	1.10	0.85	1.41	34	33.27	1.02	0.71	1.43

For those PCa cancer diagnosed prior to 1995, the overall SIR was 1.05 (95%CI: 0.98–1.12), whereas it was 1.17 (95%CI: 1.10–1.25) for those diagnosed in 1995 and later on (results not shown). Exclusion of those who have <6 months of follow-up, resulted in an overall SIR of 1.03 (95%CI: 0.97–1.08). The SIRs ranged from 1.14 (95%CI: 1.00–1.30) for lung cancer to 2.56 (95%CI: 1.32–4.47) for thyroid cancer (results not shown).

**Figure 1:**
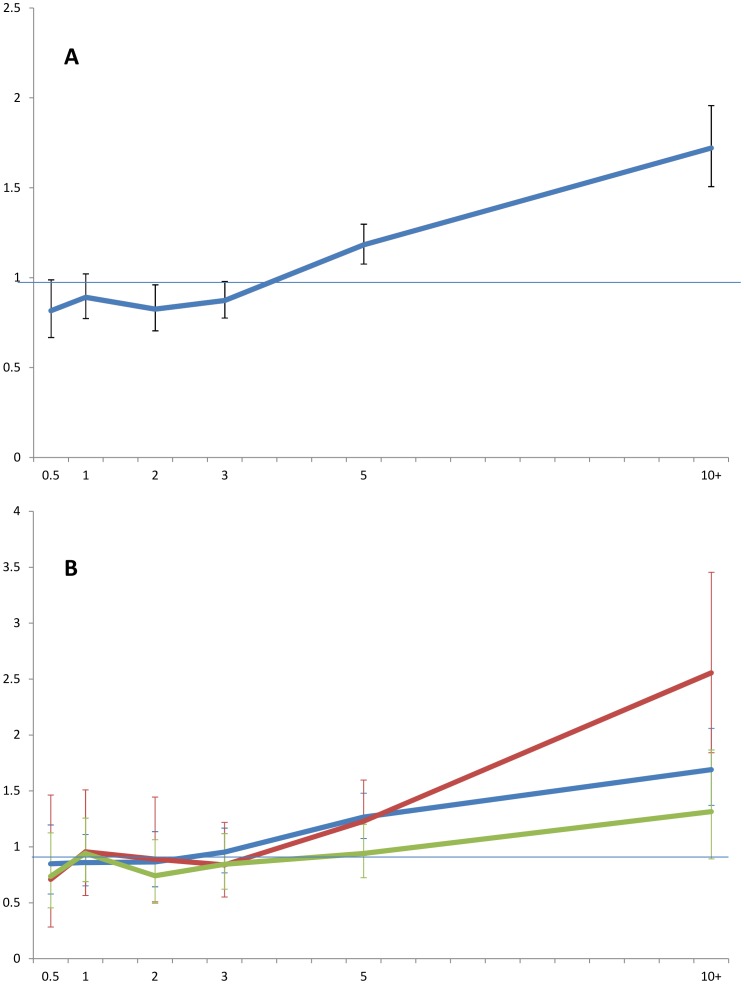
SIR and 95%CI (Y-axis) for all cancers occurring after PCa diagnosis by time (years) since time of PCa diagnosis (A), stratified by treatment (B): surgery (blue), radiotherapy (red), and androgen deprivation therapy (green).

Finally, we plotted SIRs by time since PCa diagnosis (Figure 1A) which showed a lower risk of cancer for men with PCa compared to the general population for the first four years, but then a steep increase in risk was observed. Further stratification by primary treatment (Figure 1B) illustrated an increase from 0.89 (95%CI: 0.51–1.45) at 2 years post diagnosis to 2.56 (95%CI: 1.84–3.46) at more than 10 years post diagnosis for men on radiotherapy. This increase over time was less pronounced for men undergoing surgery or ADT: 0.86 (95%CI: 0.64–1.14) to 1.69 (95%CI: 1.37–2.06) and 0.74 (95%CI: 0.50–1.07) to 1.32 (95%CI: 0.89–1.87), respectively.

## Discussion

The current study assessed in detail the risk of second primary tumours for men diagnosed with PCa in the Canton of Zurich. We found a weak increased risk for overall cancer, but a complex pattern was observed when looking at specific cancer types and PCa treatment and stage. Urological cancers were consistently more prevalent among men with PCa, irrespective of their cancer treatment or cancer stage. The increased risk of second primary tumours increased over time and was most pronounced 10 years post diagnosis for men who underwent radiotherapy.

Another Swiss study based on the Cancer Registries of the Cantons of Vaud and Neuchâtel already investigated the risk of second primary tumours in 1999. They compared men diagnosed with PCa between 1974 and 1994 with the general population and found an SIR of 0.8 (95%CI: 0.6–0.8) [18]. A more recent Swiss study based on the Geneva Cancer Registry showed that the overall SIR of secondary cancer in PCa patients treated with radiotherapy was 1.35 (p = 0.056), with elevated SIRs especially for colon cancer (4.0 (95%CI: 1.8–7.6)) [19].Our results confirm these more recent findings; however the SIR for colon cancer was only 1.40 and not statistically significant. The latter may be partly due to a smaller sample size. When we computed SIRs by study period, we observed a lower SIR of 1.05, which was not statistically significant, for cancers diagnosed between 1980 and 1995. Thus, our inconsistency with the results by Levi et al. might be due to the fact that in former times, PCa was often diagnosed at an advanced stage with shorter survival. The chance of developing a second primary tumour was lower than today. This is also consistent with our observation of the highest SIR in men diagnosed with stage I PCa, but a not statistically significant SIR among men diagnosed with advanced, i.e., stage III and stage IV, cancers.

Moreover, our results are similar compared to a Bavarian study [10], which observed that the overall risk of second primary tumours for men with PCa was significantly increased by 14% compared with the general male population. With regard to specific cancer types, a significantly increased risk was found for cancer of the bladder, kidney, pancreas, thyroid, small intestine, brain/nervous system, and melanoma of skin, leukemia, and myeloma. In the Swedish study comparing all men diagnosed with PCa between 1997–2006 and the male background population, there was also an overall increased risk of second primary tumours [8]. This study did not look at all cancer types as discussed in the current project. However, they also found an increased risk for colon, bladder, and brain cancers, as well as melanoma and non-hodgkin lymphoma. They found a lower risk for kidney cancer, but data reported in the Swedish study focused on the specific time frame of five years post PCa diagnosis, whereas in our study we have a longer follow-up. Interestingly, when investigating fatal cancer outcomes, an American study focused on men who underwent radical prostatectomy, found inverse association for all deaths due to second primary malignancies [20]. These results can however not be compared to our findings as this study by Eifler et al. focused on fatal events in a subset of men with PCa, who are likely to be healthier than the total PCa population and the total male population.

Overall, there was an increased SIR for almost all cancer types. The SIRs were especially increased for kidney, bladder, and thyroid cancer as well as non-Hodgkin's lymphoma. An increased risk of urological cancers is consistent with previous findings and most likely reflects the related increased diagnostic activity for PCa men [8].

Interestingly, stratification by treatment showed some different patterns whereby the SIRs for thyroid cancer were high for men undergoing surgery, radiotherapy, or surveillance. Similarly, non-Hodgkin's lymphoma had SIRs >2 for men undergoing surveillance and radiotherapy. In the latter group the SIR for leukemia was also of this size. The observations for men undergoing surveillance are difficult to interpret in this setting as we could not distinguish between those on surveillance and those with missing data for treatment. However, the findings for radiotherapy are consistent with findings from the American SEER database. Compared with men who received no PCa-directed radiation, men who received external beam radiation therapy were at increased risk of bladder, rectum, colon, brain, stomach, melanoma, and lung cancer [21]. As already mentioned, the lack of a statistically significant association for colon cancer may be due to the lack of statistical power.

Patterns by disease stage also need to take into account patterns by treatment as lower grade tumours are more likely to be treated curatively with surgery or radiotherapy. Nevertheless, the SIRs for urological cancers were consistent across grade and only slightly higher for those undergoing radical prostatectomy; again confirming the increased diagnostic activity for PCa men. Furthermore, stratification by treatment as well as time since diagnosis confirmed previous findings by Murray et al. [22]. The overall SIR for men on radiotherapy was 1.19, but increased to 2.56 for men followed up for more than 10 years, whereas this increase over time was much less pronounced for men who underwent surgery or ADT.

With respect to aetiological mechanisms, our analysis for those cancers thought to be associated with IGF-1 showed a consistent increased risk, also when stratified by treatment. Only for men on ADT, there was no difference with the background population. Men on ADT most likely had a more severe tumour and thus lower life expectancy, which is also reflected in the analysis for IGF-1 related cancer stratified by tumour stage. This observational study cannot claim causality, but it supports further evidence for the hypothesis that the IGF-1 pathway is involved in development of epithelial cancers, as was also demonstrated in a study for second primary tumours in patients with a head and neck cancer [23]. Higher IGF-1 levels were associated with and increased risk of second primary tumours with a hazard ratio of 2.78 (95%CI: 1.62–4.77).

Coverage of the Registry is almost complete, though there is some under-registration due to insufficient legal regulation of cancer registration on cantonal level [11]. Under-registration of PCa cases due to limited access to two pathology institutes (4–10%), however, only occurred between 2007 and 2010 and, thus, only affected the most recent years of our analysis. Another limitation is that we only have information on “radiotherapy” and we cannot distinguish between external beam radiation therapy (EBRT) and brachytherapy. Only during the last few years the Cancer Registry started collected more detailed treatment information (i.e. transition from curative to palliative treatment) so that it was not possible to make further distinctions between treatment pathways. We only collected information from time of diagnosis. Moon et al. have previously shown that patients receiving EBRT had a higher odds of developing cancer compared with men receiving no radiation therapy, but the odds was not increased for men receiving radioactive implants or isotopes [21]. However, not all studies confirm this difference [5].

### Conclusions

The current analysis showed a detailed assessment of risk of second primary tumours following PCa diagnosis. There was a clear increased risk, but patterns by disease stage and treatment were complex and varied for different tumour types. Overall, the findings illustrate the need for careful management of men with PCa as they are more prone to develop another primary tumour.

## References

[1] American Cancer Society (2013) Prostate Cancer.

[2] Herrmann C, Cerny T, Savidan A, Vounatsou P, Konzelmann I, et al (2013) Cancer survivors in Switzerland: a rapidly growing population to care for. BMC Cancer 13: 287. 23764068 10.1186/1471-2407-13-287 PMC3685597

[3] Wallner LP, Wang R, Jacobsen SJ, Haque R (2013) Androgen deprivation therapy for treatment of localized prostate cancer and risk of second primary malignancies. Cancer Epidemiol Biomarkers Prev 22: 313–316. 23292083 10.1158/1055-9965.EPI-12-1137 PMC3758254

[4] Nash GF, Turner KJ, Hickish T, Smith J, Chand M, et al (2012) Interactions in the aetiology, presentation and management of synchronous and metachronous adenocarcinoma of the prostate and rectum. Ann R Coll Surg Engl 94: 456–462. 23031761 10.1308/003588412X13373405384611 PMC3954237

[5] Zelefsky MJ, Pei X, Teslova T, Kuk D, Magsanoc JM, et al (2012) Secondary cancers after intensity-modulated radiotherapy, brachytherapy and radical prostatectomy for the treatment of prostate cancer: incidence and cause-specific survival outcomes according to the initial treatment intervention. BJU Int 110: 1696–1701. 22889401 10.1111/j.1464-410X.2012.11385.x

[6] Zelefsky MJ, Housman DM, Pei X, Alicikus Z, Magsanoc JM, et al (2012) Incidence of secondary cancer development after high-dose intensity-modulated radiotherapy and image-guided brachytherapy for the treatment of localized prostate cancer. Int J Radiat Oncol Biol Phys 83: 953–959. 22172904 10.1016/j.ijrobp.2011.08.034

[7] Hinnen KA, Schaapveld M, van Vulpen M, Battermann JJ, van der Poel H, et al (2011) Prostate brachytherapy and second primary cancer risk: a competitive risk analysis. J Clin Oncol 29: 4510–4515. 22025166 10.1200/JCO.2011.35.0991

[8] Van Hemelrijck M, Drevin L, Holmberg L, Garmo H, Adolfsson J, et al (2012) Primary cancers before and after prostate cancer diagnosis. Cancer 118: 6207–6216. 22674346 10.1002/cncr.27672

[9] Margel D, Baniel J, Wasserberg N, Bar-Chana M, Yossepowitch O (2011) Radiation therapy for prostate cancer increases the risk of subsequent rectal cancer. Ann Surg 254: 947–950. 22107741 10.1097/SLA.0b013e3182382fd5

[10] Braisch U, Meyer M, Radespiel-Troger M (2012) Risk of subsequent primary cancer among prostate cancer patients in Bavaria, Germany. Eur J Cancer Prev 21: 552–559. 22433633 10.1097/CEJ.0b013e328351c748

[11] Dehler S, Korol D, Prater J, Laue R, Morf S, et al.. (2012) Krebsregister der Kantone Zurich und Zug.

[12] Foundation National Institue for Cancer Epidemiology and Registrationm (2014) Cancer Incidence and Mortality in Switzerland by NICER: Data and Methods.

[13] European Commission Eurostat (2013) European shortlist of causes of death.

[14] Breslow N, Day N (1987) Statistical methods in cancer research. Volume II - The design and analysis of cohort studies. 406 p. 3329634

[15] Zar N, Garmo H, Holmberg L, Hellman P (2008) Risk of second primary malignancies and causes of death in patients with adenocarcinoma and carcinoid of the small intestine. Eur J Cancer 44: 718–725. 18207733 10.1016/j.ejca.2007.12.003

[16] Smith GD, Gunnell D, Holly J (2000) Cancer and insulin-like growth factor-I. A potential mechanism linking the environment with cancer risk. BMJ 321: 847–848. 11021847 10.1136/bmj.321.7265.847 PMC1118665

[17] Pawlish KS, Schottenfeld D, Severson R, Montie JE (1997) Risk of multiple primary cancers in prostate cancer patients in the Detroit metropolitan area: a retrospective cohort study. Prostate 33: 75–86. 9316648 10.1002/(sici)1097-0045(19971001)33:2<75::aid-pros1>3.0.co;2-l

[18] Levi F, Randimbison L, Te VC, Erler G, La Vecchia C (1999) Second primary tumors after prostate carcinoma. Cancer 86: 1567–1570. 10526286 10.1002/(sici)1097-0142(19991015)86:8<1567::aid-cncr25>3.0.co;2-z

[19] Rapiti E, Fioretta G, Verkooijen HM, Zanetti R, Schmidlin F, et al (2008) Increased risk of colon cancer after external radiation therapy for prostate cancer. Int J Cancer 123: 1141–1145. 18546265 10.1002/ijc.23601

[20] Eifler JB, Humphreys EB, Agro M, Partin AW, Trock BJ, et al (2012) Causes of death after radical prostatectomy at a large tertiary center. J Urol 188: 798–801. 22819416 10.1016/j.juro.2012.04.109

[21] Moon K, Stukenborg GJ, Keim J, Theodorescu D (2006) Cancer incidence after localized therapy for prostate cancer. Cancer 107: 991–998. 16878323 10.1002/cncr.22083

[22] Murray L, Henry A, Hoskin P, Siebert FA, Venselaar J, et al.. (2014) Second primary cancers after radiation for prostate cancer: A systematic review of the clinical data and impact of treatment technique. Radiother Oncol. 10.1016/j.radonc.2013.12.012 PMC3988985 24485765

[23] Wu X, Zhao H, Do K, Johnson M, Dong Q, et al (2004) Serum levels of insulin growth factor (IGF-I) and IGF-binding protein predict risk of second primary tumors in patients with head and neck cancer. Clin Cancer Res 10: 3988–3995. 15217929 10.1158/1078-0432.CCR-03-0762

